# Hereditary pheochromocytoma/paraganglioma syndrome with a novel mutation in the succinate dehydrogenase subunit B gene in a Japanese family: two case reports

**DOI:** 10.1186/s13256-021-02852-z

**Published:** 2021-05-22

**Authors:** Rei Hirose, Yuya Tsurutani, Chiho Sugisawa, Kosuke Inoue, Sachiko Suematsu, Maki Nagata, Naoki Hasegawa, Yukio Kakuta, Masato Yonamine, Kazuhiro Takekoshi, Noriko Kimura, Jun Saito, Tetsuo Nishikawa

**Affiliations:** 1 Endocrinology and Diabetes Center, Yokohama Rosai Hospital, 3211 Kozukue-cho, Kouhoku-ku, Yokohama, Kanagawa 222-0036 Japan; 2 Department of Epidemiology, UCLA Fielding School of Public Health, 650 Charles E. Young Dr. South, 16-035 Center for Health Sciences, Los Angeles, CA USA; 3 Department of Urology, Yokohama Rosai Hospital, 3211 Kozukue-cho, Kouhoku-ku, Yokohama, Kanagawa 222-0036 Japan; 4 Department of Pathology, Yokohama Rosai Hospital, 3211 Kozukue-cho, Kouhoku-ku, Yokohama, Kanagawa 222-0036 Japan; 5 Laboratory of Laboratory/Sports Medicine, Division of Clinical Medicine, Faculty of Medicine, University of Tsukuba, 1-1-1 Tennodai, Tsukuba, Ibaraki 305-8577 Japan; 6 Department of Diagnostic Pathology, National Hospital Organization Hakodate Hospital, 18-16 Kawahara-cho, Hakodate, Hokkaido 041-8512 Japan

**Keywords:** Hereditary pheochromocytoma/paraganglioma syndrome, Succinate dehydrogenase subunit B, Metastatic paraganglioma, Surveillance

## Abstract

**Background:**

Pheochromocytoma and paraganglioma caused by succinate dehydrogenase gene mutations is called hereditary pheochromocytoma/paraganglioma syndrome. In particular, succinate dehydrogenase subunit B mutations are important because they are strongly associated with the malignant behavior of pheochromocytoma and paraganglioma . This is a case report of a family of hereditary pheochromocytoma/paraganglioma syndrome carrying a novel mutation in succinate dehydrogenase subunit B.

**Case presentation:**

A 19-year-old Japanese woman, whose father died of metastatic paraganglioma, was diagnosed with abdominal paraganglioma, and underwent total resection. Succinate dehydrogenase subunit B genetic testing detected a splice-site mutation, c.424-2delA, in her germline and paraganglioma tissue. Afterwards, the same succinate dehydrogenase subunit B mutation was detected in her father’s paraganglioma tissues. *In silico* analysis predicted the mutation as “disease causing.” She is under close follow-up, and no recurrence or metastasis has been observed for 4 years since surgery.

**Conclusions:**

We detected a novel succinate dehydrogenase subunit B mutation, c.424-2delA, in a Japanese family afflicted with hereditary pheochromocytoma/paraganglioma syndrome and found the mutation to be responsible for hereditary pheochromocytoma/paraganglioma syndrome. This case emphasizes the importance of performing genetic testing for patients with pheochromocytoma and paraganglioma suspected of harboring the succinate dehydrogenase subunit B mutation (that is, metastatic, extra-adrenal, multiple, early onset, and family history of pheochromocytoma and paraganglioma) and offer surveillance screening to mutation carriers.

## Background

Pheochromocytoma (PCC) and paraganglioma (PGL) are rare neuroendocrine tumors arising from the chromaffin cells of the adrenal medulla and extra-adrenal autonomic paraganglia, respectively. Up to 40% of patients with PCC/PGL (PPGL) carry a germline mutation [1]. To date, over 19 types of susceptibility genes for PPGL, including *SDHA*, *SDHB*, *SDHC*, *SDHD*, *RET*, *VHL*, *NF1*, *TMEM127*, and *MAX* have been reported [2]. Of these, succinate dehydrogenase (SDH)-related PPGLs are called hereditary pheochromocytoma/paraganglioma syndrome (HPPS).

In particular, *SDHB* mutations are important because they are highly associated with the malignant behavior of PPGL [3–7]. *SDHB* encodes one of four subunits of mitochondrial complex II (SDH). Consistent with Knudson’s two-hit hypothesis, the function of complex II is lost by biallelic inactivation (typically resulting from one inherited and one somatic event) of *SDHB*, resulting in increased reactive oxygen species levels, hypoxia-inducible factor-α (HIF-1α) activation, and pseudohypoxia, which promote tumor formation and catecholamine oversecretion [3, 8–12]. Therefore, patients with HPPS should be actively tested for *SDHB* mutations, and information on a novel mutation should be reported. Herein, we report a family of HPPS with a novel *SDHB* mutation, c.424-2delA.

## Case presentation

**Fig. 1:**
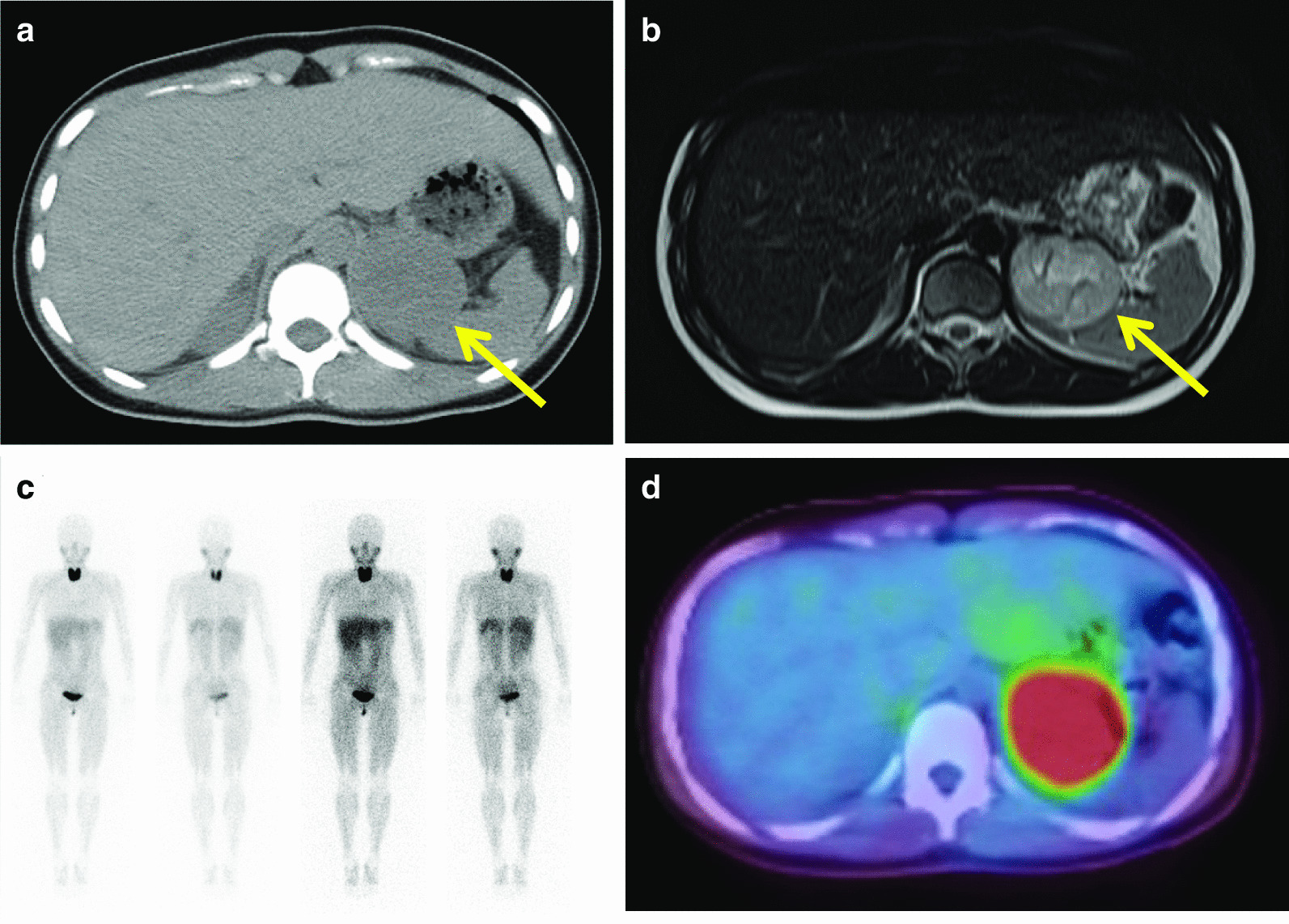
Abdominal computed tomography scan showing a 50 mm mass above the left kidney (arrow) (**a**). Abdominal MRI showing the mass is nonhomogeneous and of moderate intensity on T2-weighted images (arrow) (**b**). ^123^I-MIBG scintigraphy showing no accumulation in the mass (**c**). FDG-PET showing accumulation (SUVmax 10.9) in the mass (**d**). *CT*, computerized tomography; *FDG-PET*, fluorodeoxyglucose-positron emission tomography; *MIBG*, metaiodobenzylguanidine; *MRI*, magnetic resonance imaging; *SUVmax*, maximum standardized uptake value

**Fig. 2:**
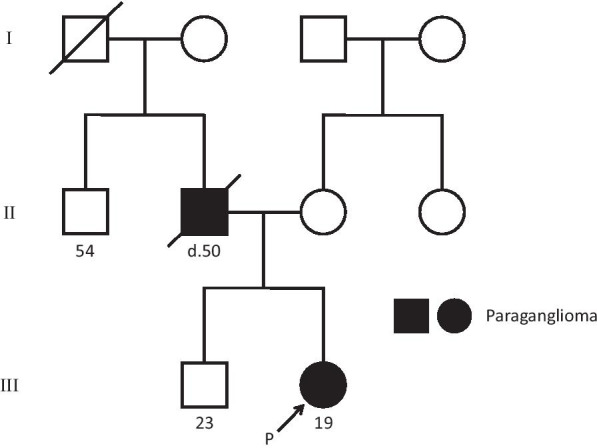
Pedigree and clinical phenotype of family member

Patient 1. Daughter (III-2, proband): A 19-year-old Japanese woman was diagnosed with hypertension (200/140 mmHg) at a physical examination. The following month, she visited an emergency hospital with complaints of palpitation and dyspnea. Abdominal computed tomography (CT) revealed a 50 mm mass above the left kidney (Fig. 1a). Her blood catecholamine levels were elevated. Her father had died owing to multiple metastases of PGL when he was 50 years old (Fig. 2). She was suspected of having PPGL and was referred to our hospital for further examination.

The patient’s consciousness was clear. A physical examination revealed the following findings: body temperature, 37.4 °C; blood pressure, 115/73 mmHg; and pulse rate, 95 bpm. She was 152 cm in height with a body weight of 48 kg (body mass index, 20.8 kg/m^2^). Hypertensive fundus changes (H1S1/H1S1) were observed. Heart sound was regular, and a Levine II/VI systolic murmur was heard along the left sternal border. The abdomen was flat and soft, and the mass was not palpable.

The patient’s laboratory data are presented in Table 1, which indicates excessive catecholamine production. Abdominal magnetic resonance imaging (MRI) showed that the mass was nonhomogeneous and moderate in intensity on T2-weighted images (Fig. 1b). ^123^I-metaiodobenzylguanidine (MIBG) scintigraphy showed no accumulation in the mass (Fig. 1c). ^18^F-fluorodeoxyglucose-positron emission tomography (FDG-PET) showed accumulation (maximum standardized uptake value 10.9) in the mass (Fig. 1d). Based on these results, she was diagnosed with left PCC or abdominal PGL. She was prescribed doxazosin (up to 20 mg/day) preoperatively for alpha blockade.

**Table 1:** Laboratory data of the daughter on admission

Parameter	Value	Unit	Reference range	Parameter		Value	Unit	Reference range
Endocrinological test				Urinary analysis				
Adrenaline	< 0.01	ng/mL	0–0.17	Adrenaline	3.9		µg/day	1.1–22.5
Noradrenaline	3.6	ng/mL	0.15–0.57	Noradrenaline	2267		µg/day	29.2–118
Dopamine	0.13	ng/mL	0–0.03	Dopamine	1053		µg/day	100–1000
ACTH	16.2	pg/mL	7.2–63.3	Metanephrine	0.057		mg/day	0.05–0.2
Cortisol	6.6	µg/dL	4.0–19.3	Normetanephrine	4.7		mg/day	0.1–0.28
PRA	2.6	ng/mL/hour	0.3–2.9	Cortisol	36.9		µg/day	11.2–80.3
PAC	149	pg/mL	29.9–159	Aldosterone	5.7		µg/day	0–10
TSH	1.92	µIU/mL	0.500–5.000					
FT3	3.1	pg/mL	2.3–4.3					
FT4	1.2	ng/dL	0.9–1.7					
iPTH	58.1	pg/mL	10–65					
Calcitonin	< 0.50	pg/mL	< 6.40					

*ACTH*, adrenocorticotropic hormone; *PRA*, plasma renin activity; *PAC*, plasma aldosterone concentration; *TSH*, thyroid-stimulating hormone; *FT3*, free triiodothyronine; *FT4*, free thyroxine; *iPTH*, intact parathormone

**Fig. 3:**
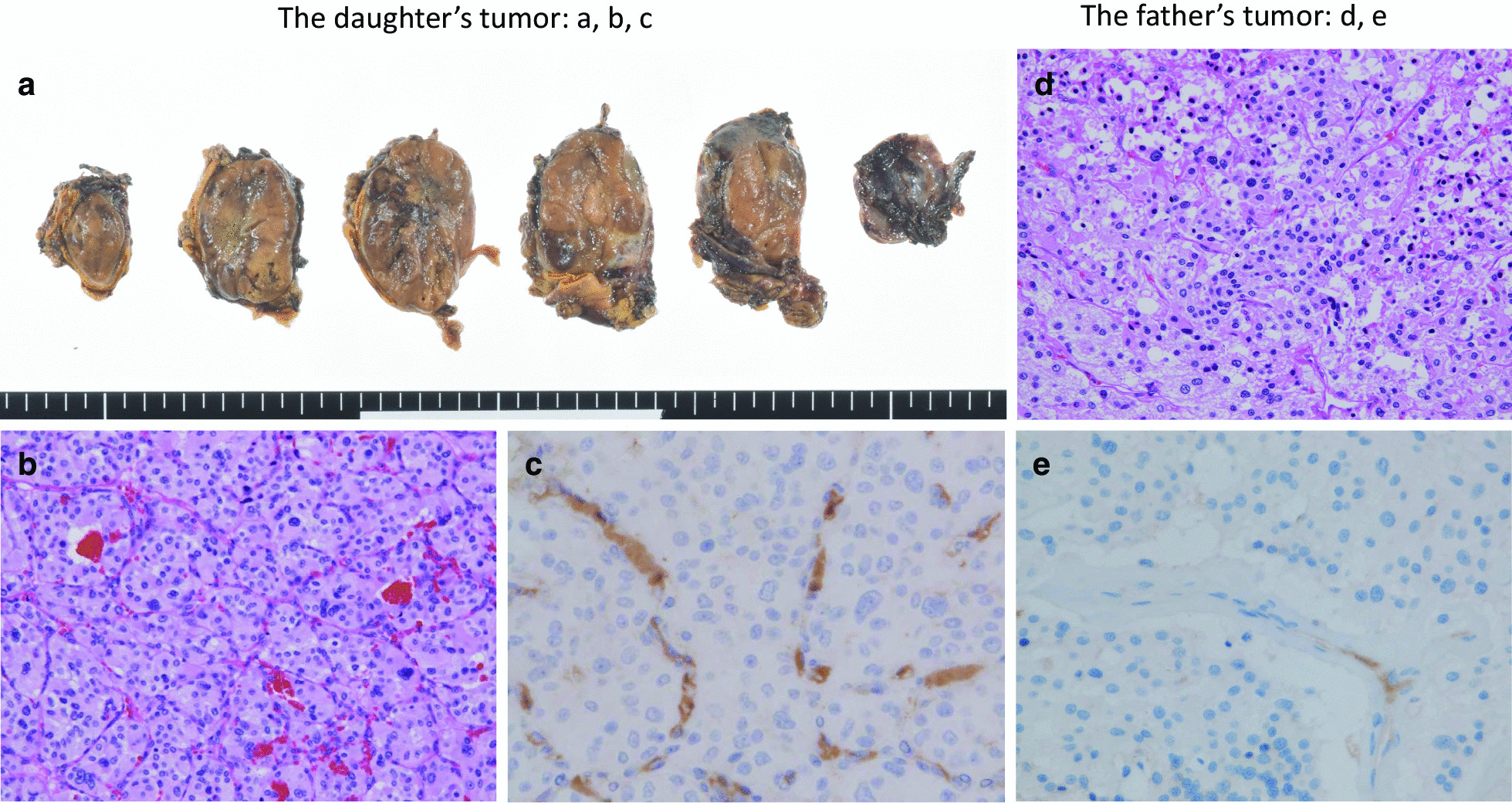
The tumors of the daughter (**a**–**c**), and of the father (**d**, **e**). **a **Gross section of the PGL. The tumor locates beneath the adrenal gland. **b** The tumor shows high cellularity with a zellballen pattern. GAPP score is 6 points. **c** SDHB immunohistochemistry is negative in tumor cells; only endothelial cells are positive. **d** Tumor cells arranged in zellballen pattern. Intracytoplasmic vacuoles are degenerative changes due to chemotherapy. **e** SDHB immunohistochemistry is negative in tumor cells, same as in **c**. **b** and **d**: ×200 magnification, and **c** and **e**: ×400 magnification. *GAPP*, grading system for adrenal pheochromocytoma and paraganglioma; *PGL*, paraganglioma; *SDHB*, succinate dehydrogenase subunit B

**Fig. 4:**
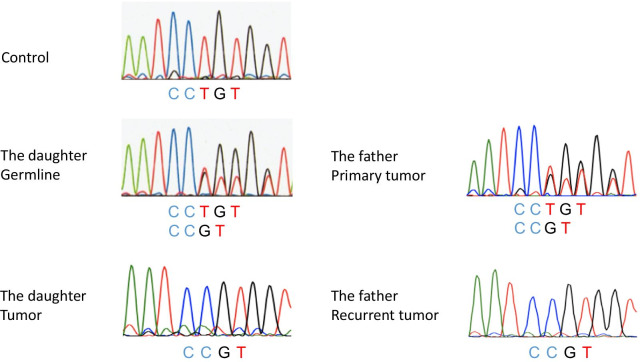
Results of a PCR-direct sequencing of *SDHB* intron 4/exon 5 junction. For the daughter, the peripheral blood DNA (germline) showed c.424-2delA in heterozygosity. The tumor tissue DNA showed loss of the wild-type T allele, suggesting LOH. For the father, the primary tumor tissue DNA showed c.424-2delA in heterozygosity. The recurrent tumor tissue DNA showed loss of the wild-type T allele, suggesting LOH. *LOH*, loss of heterozygosity; *PCR*, polymerase chain reaction; *SDHB*, succinate dehydrogenase subunit B

She underwent a laparoscopic left adrenalectomy. The weight of the tumor mass was 48.5 g (50 × 40 × 25 mm). A pathological investigation confirmed the diagnosis of retroperitoneal PGL (Fig. 3 a, b). The grading system for adrenal pheochromocytoma and paraganglioma (GAPP) score was 6 points, suggesting a tumor with intermediate-grade malignancy [13]. Immunohistochemical (IHC) staining of SDHA yielded positive results, and IHC staining of SDHB yielded negative results, suggesting an *SDHB*, *SDHC*, or *SDHD* mutation [14, 15] (Fig. 3c). We performed genetic testing for *SDHB* with written consent from the patient and her mother. We extracted the germline DNA from her peripheral blood and screened the eight exons of the *SDHB* gene. The results of polymerase chain reaction (PCR)-direct sequencing method, conducted as described previously [16], showed that she had heterozygous germline mutations in the *SDHB* intron 4/exon 5 junction (c.424-2delA) (Fig. 4). This mutation is predicted to constitute a splice acceptor site using splice site prediction [17] and NetGene2 [18]. Therefore, it can lead to missplicing, such as exon skipping, activation of a cryptic splice site, or intron retention, resulting in the production of abnormal proteins [19]. In addition, *in silico* analysis, using MutationTaster [20], predicted this mutation was “disease causing.” Sequencing analysis of the PGL tissue showed loss of the wild-type T allele, suggesting loss of heterozygosity (LOH) (Fig. 4). After surgery, her blood pressure normalized, and the plasma and urinary catecholamine levels were within the normal range. We are performing a close follow-up with biannual medical examinations, biochemical tests, MRI of the abdomen, and an annual MRI from the neck to the pelvis. No recurrence or metastasis has been observed 4 years since surgery.

Patient 2. Father (II-2): A 40-year-old Japanese man (father of patient 1) had back pain and was examined by MRI 11 years before the first visit of the daughter. An abdominal mass with low intensity on the T1-weighted image and high intensity on the T2-weighted image, and a mass on the seventh rib, were found. An endocrinological examination showed that dopamine in a 24-hour urine collection was as high as 1006.3 μg/day. Adrenaline and noradrenaline levels were within the normal range. ^123^I-MIBG scintigraphy showed no accumulation. He was considered to have a retroperitoneal tumor and metastasis to the rib. The patient underwent total resection of the retroperitoneal tumor and was pathologically diagnosed with retroperitoneal PGL (10 × 7 × 4 cm) (Fig. 3d). Although chemotherapy with cyclophosphamide, vincristine, and dacarbazine, and radiation therapy for rib metastasis, were performed, tumor recurrence in the pelvis was found when he was 46 years old. The tumor was resected and diagnosed pathologically as recurrent retroperitoneal PGL (55 × 50 mm). Later, hepatic metastasis and pleural dissemination were observed, and partial lobectomy of the liver and ^131^I-MIBG therapy were performed. However, the treatment was not effective, and he passed away when he was 50 years old.

After the daughter was diagnosed with PGL, we added a further study to the surgical specimen of the primary and recurrent PGL of the father. The GAPP score of the primary PGL was 3 points, suggesting the tumor was an intermediate-grade malignancy [13]. IHC staining of SDHB of the primary PGL was negative (Fig. 3e). *SDHB* genetic testing revealed that both primary and recurrent PGLs harbored the mutation c.424-2delA, which is the same as the PGL of the daughter (Fig. 4). The primary PGL tissue DNA showed c.424-2delA in heterozygosity, whereas the recurrent PGL tissue DNA showed loss of the wild-type T allele, suggesting LOH.

Although our two cases had the same *SDHB* mutation, phenotypes such as the age of onset, biochemical phenotype, and metastasis were different (Table 2). The son of patient 2 (III-1) might have the same *SDHB* mutation, given his family history; however, he did not provide consent to undergo genetic testing.

**Table 2:** Clinical characteristics of two cases

	Daughter	Father
Sex	Female	Male
Age at diagnosis	19	40
Primary tumor size	5 × 4 × 2.5 cm	10 × 7 × 4 cm
Primary tumor localization	Abdominal PGL	Abdominal PGL
Catecholamine type	Noradrenaline type	Nonfunctioning type
Metastasis	No metastasis	Rib, liver, pleura
Ki-67 index	5.10%	1.50%
GAPP score	6 (moderately differentiated)	3 (moderately differentiated)

*PGL*, paraganglioma; *GAPP*, grading system for adrenal pheochromocytoma and paraganglioma

## Discussion and conclusions

We encountered a family of HPPS with a novel *SDHB* splice-site mutation (c.424-2delA). The father died of metastatic PGL, and the daughter is under close follow-up, after total resection of the retroperitoneal PGL.

As the *SDHB* mutation is strongly associated with the malignant behavior of PPGL, performing genetic testing in patients with PPGL suspected of harboring *SDHB* mutations is important. Identification of a novel disease causing *SDHB* mutation may contribute to expanding our knowledge about PPGL and facilitate more patient-tailored management. To date, 289 unique *SDHB* mutations have been described [21]. Most germline mutations are randomly distributed over *SDHB*, and at least two hot spots have been described [22–24]. Germline *SDHB* mutations are inherited in an autosomal dominant manner, and their penetrance is approximately 20% by age 50 years and 40% by age 70 years [24–26]. The phenotype of PPGL with *SDHB* mutation is often characterized as metastatic, with extra-adrenal development (especially abdominal PGL), and multiple and early onset [3, 14]. Therefore, *SDHB* genetic testing should be performed in patients with PPGL who have these characteristics or a family history of PPGL. In the present cases, as the daughter had early-onset abdominal PGL with a family history of metastatic PGL, *SDHB* mutation was strongly suspected. In addition, a false-negative ^123^I-MIBG scintigraphy, as observed in the daughter, is frequently associated with *SDHB* mutations [27]. *SDHB* genetic testing identified the novel splice-site mutation c.424-2delA in her germline, PGL tissue*,* and her father’s primary and recurrent PGL tissue*. In silico* analysis predicted this mutation was “disease causing.” As for her PGL and the recurrent PGL of her father, LOH of *SDHB* was suggested, which indicated tumor formation by biallelic inactivation of *SDHB*. To the best of our knowledge, this is the first report showing that the *SDHB* mutation c.424-2delA is responsible for PPGL [21, 28].

Multiple cases of different phenotypes within the same family with the same *SDHB* mutation have been reported [4, 16, 29]. These different phenotypes may be due to the involvement of several disease-related genes and the variation in epigenetic changes. Many reports have indicated there is no genotype–phenotype correlation in the *SDHB* mutant PPGL [23, 25, 30]. However, Andrews *et al.* reported that a specific missense mutation in *SDHB* (p.Ile127Ser) could have a marked effect on protein structure, resulting in increased penetrance and risk of PPGL [31], suggesting the possibility of genotype–phenotype correlation in *SDHB* mutation. Further investigation of genotype–phenotype correlation may advance our knowledge about individual treatment and surveillance screening based on genotype.

Our cases indicate the importance of surveillance screening in *SDHB* mutation carriers, although there is no widely agreed consensus regarding optimal surveillance for asymptomatic carriers and those in whom the presenting tumor has been resected. Surveillance screening in *SDHB* mutation carriers enables early detection and timely resection of *SDHB*-associated tumors, reducing the risk of metastatic disease [32]. In our cases, if *SDHB* genetic testing had been performed on the father with metastatic PGL, and *SDHB* mutation had been identified, surveillance screening might have revealed the PGL in the daughter before it became secretory and/or symptomatic. The Endocrine Society recommends lifelong follow-up for all patients with PPGL [33]. The European Society of Endocrinology recommends follow-up for at least 10 years after surgery in all patients with PPGL and lifelong follow-up, especially for patients at high risk of recurrence (young patients and those with a genetic disease, a large tumor, and/or a PGL) [34]. However, there is no official guideline for the surveillance screening of patients with *SDHB* mutations. Some reported ideas of surveillance screening in *SDHB* mutation carriers are presented in Table 3 [4, 32, 35–37]. We are performing a close follow-up on the daughter, considering she has developed PGL and her father had taken an aggressive course. All first-degree relatives are recommended to undergo targeted *SDHB* genetic testing of the proband’s mutation with genetic counseling; however, children should be offered testing if they are recommended surveillance [36, 37]. We recommended targeted *SDHB* genetic testing to her older brother (III-1), but he did not provide consent to undergo genetic testing.

**Table 3:** Summary of reported ideas of surveillance screening for patients with *SDHB* mutation

Refs.	Year	Clinical review (physical examination, BP, PR)	Biochemical test	Imaging test	Age to start surveillance (years)
[4]	2006	Frequency not stated	Annual	MRI or CT from neck to pelvis every 2 years Consider ^18^F-DOPA-PET	10
[35]	2014	Every 6–12 months	Annual	MRI or CT from thorax to pelvis every 6–24 months MRI or CT of skull base and neck every 2–4 years MIBG scintigraphy every 2–4 years	5–10
[32]	2019	Annual	Annual	MRI of abdomen every year MRI from skull base to pelvis every 2 years	5
[36]	2019	Annual	Annual	MRI from skull base to pelvis every 2–3 years	5 (10 as to MRI)
[37]	2019	Every 6 months	Annual	Rapid whole-body MRI every 2 years	5 years before the earliest age of onset in the family

*BP*, blood pressure; *PR*, pulse rate; *MRI*, magnetic resonance imaging; *CT*, computed tomography; *MIBG*, metaiodobenzylguanidine; *18F-DOPA-PET*, 6-[18F]-fluoro-L-3,4-dihydroxyphenylalanine positron emission tomography

In conclusion, we detected a novel *SDHB* mutation, c.424-2delA, in a Japanese family afflicted with HPPS and found the mutation to be responsible for HPPS. Since *SDHB* mutation is associated with the malignant behavior of PPGL, it is important to perform genetic testing for patients suspected of harboring the *SDHB* mutation (that is, metastatic, extra-adrenal, multiple, early onset, and family history of PPGL) and offer surveillance screening to mutation carriers.

## Data Availability

Data sharing is not applicable to this article, as no datasets were generated or analyzed during the current study.

## Abbreviations

PPGL: Pheochromocytoma and paraganglioma
HPPS: Hereditary pheochromocytoma/paraganglioma syndrome
SDHB: Succinate dehydrogenase subunit B
PCC: Pheochromocytoma
PGL: Paraganglioma
SDH: Succinate dehydrogenase
HIF-1α: Hypoxia-inducible factor-α
CT: Computed tomography
MRI: Magnetic resonance imaging
MIBG: Metaiodobenzylguanidine
FDG-PET: ^18^F-fluorodeoxyglucose-positron emission tomography
GAPP: Grading system for adrenal pheochromocytoma and paraganglioma
IHC: Immunohistochemistry
PCR: Polymerase chain reaction
LOH: Loss of heterozygosity
